# GroEL-Assisted Protein Folding: Does It Occur Within the Chaperonin Inner Cavity?

**DOI:** 10.3390/ijms10052066

**Published:** 2009-05-12

**Authors:** Victor V. Marchenkov, Gennady V. Semisotnov

**Affiliations:** Institute of Protein Research, Russian Academy of Sciences, 142290, Russian Federation, Pushchino, Moscow Region, Institutskaya street, 4, Russia; E-Mail: march@phys.protres.ru

**Keywords:** chaperones, GroEL/ES chaperonin system, protein folding, protein aggregation

## Abstract

The folding of protein molecules in the GroEL inner cavity under the co-chaperonin GroES lid is widely accepted as a crucial event of GroEL-assisted protein folding. This review is focused on the data showing that GroEL-assisted protein folding may proceed out of the complex with the chaperonin. The models of GroEL-assisted protein folding assuming ligand-controlled dissociation of nonnative proteins from the GroEL surface and their folding in the bulk solution are also discussed.

## 1. Introduction

*In vitro* experimental studies of protein unfolding and refolding reactions have led to an important conclusion that necessary and sufficient information on the spatial structure of proteins is included in their amino acid sequences [1,2]. However, the study of protein creation *in vivo* has revealed a number of cellular protein factors that are vital for the formation of the protein native conformation [3,4]. These protein factors, known as molecular chaperones [3], provide optimal conditions for protein folding in the cell which mostly prevent aggregation of nonfolded protein molecules. Besides, some chaperones participate in trans-membrane transport and degradation of proteins [5–8]. Many chaperones are members of a large group of heat shock proteins (hsp), whose biosynthesis in the cell is in effect enhanced by various cell stresses [9]. A number of chaperones are homo- or heterooligomers, consisting of many subunits usually combined in ring-like (toroidal) quaternary structures. Such complex quaternary structures are characteristic, for example, of hsp60 chaperones (chaperonins) and some representatives of small heat shock proteins [10–13].

**Figure 1.**
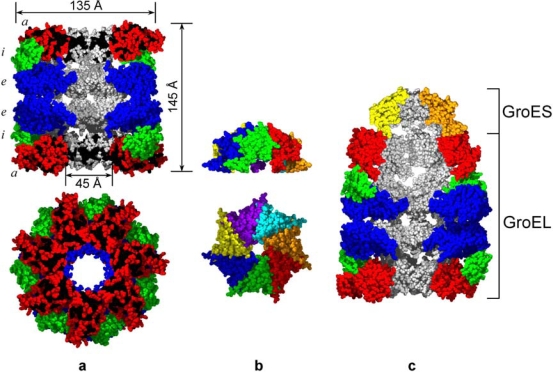
Crystal structures of (a) GroEL chaperonin, (b) co-chaperonin GroES and (c) their complex. To show the inner cavity of GroEL (a) and “Anfinsen’s cage” formed by the GroEL – GroES complex (c) front subunits of each GroEL ring and GroES have been removed. GroEL subunit domains (*a*-apical, *i*-intermediate, and *e*-equatorial) and GroES subunits are shown by different colors. Black color shows exposed hydrophobic residues on the top of apical domains. The figure was plotted using the SwissPdb Viewer [109] and POV-Ray (www.povray.org) freeware and files 1OEL [20,21] and 1AON [32] which are available in PDB.

Many studies have concentrated on the structural and functional properties of chaperonins, which assist protein folding in an ATP-dependent manner [10–13]. The GroE chaperonin system from *Escherichia coli*, consisting of two homooligomeric proteins GroEL and GroES, has been the object of numerous experimental and theoretical studies (see e.g. [12,14–32]). This system assists folding of various proteins through multiple rounds of binding and release of protein targets both *in vivo* and *in vitro*. GroEL shows no pronounced specificity towards nonnative protein targets and binds 40% of proteins from denatured extract of *E. coli* cells [31] and 30% of newly synthesized proteins [24,25]. The electron microscopy [29] and crystallographic [20, 21] data show that GroEL consists of 14 identical subunits of 57 kDa each. The subunits are arranged into two stacked heptameric toroids, constituting a cylinder of 145 Å height and 135 Å diameter, with a central inner channel of 45 Å in diameter (Figure 1).

**Figure 2.**
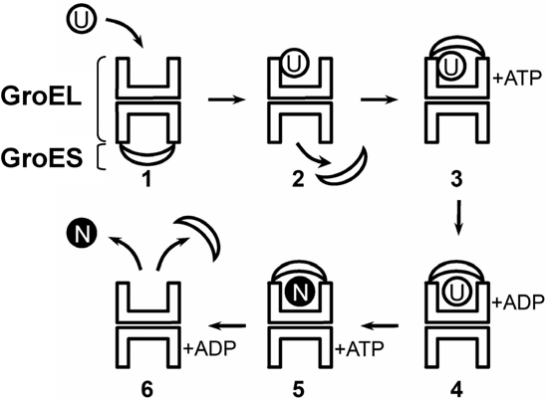
Scheme illustrating the mechanism of GroEL/ES assisted protein folding in the inner cavity (“Anfinsen’s cage”). Non-native (unfolded) and native conformations of protein target are indicated as U and N, respectively.

Each subunit of GroEL is composed of three clearly defined domains: apical (a), intermediate (i), and equatorial (e). The apical domains (191 – 376 amino acid residues) provide the binding sites for nonnative protein targets and co-chaperonin GroES [19,22,23,27,28]. The data from electron microscopy show that the nonnative protein target is bound at the edge of the inner channel [19,22,27,28]. At the same time the end-surfaces of the GroEL cylinder contain many exposed hydrophobic clusters (Figure 1) which may serve as additional sites for fixation of target proteins on the GroEL surface. The equatorial domain (6 – 133 and 409 – 523 amino acid residues) is the largest domain of the GroEL subunit and provides the majority of intersubunit and interring contacts. The coordinates of 25 C- and 5 N-terminal amino acid residues of the GroEL subunit are not resolved by crystallography probably because of the high mobility of the residues [20]. However the data from electron microscopy and neutron diffuse scattering show that these terminal amino acid residues are located in the central channel of GroEL at the level of equatorial domains [22,27,28,30]. The ATP/ADP binding site is in the upper part of the equatorial domain on the inner surface of the central channel [18]. The intermediate domain of the GroEL subunit contains 89 amino acid residues (134 – 190 and 377 – 408) and connects the equatorial and apical domains providing large-scale conformational changes of the GroEL particle when interacting with ligands [18,22,32]. Co-chaperonin GroES consists of 7 identical subunits arranged in a dome-like quaternary structure of 30 Å height, 70–80 Å in diameter and with a hole of 10 Å in diameter (Figure 1) [26]. In the presence of Mg-ATP or Mg-ADP, GroES interacts with GroEL inducing large scale movements of the apical and intermediate domains due to which the extensive inner cavity chamber is formed under the GroES lid (Figure 1) [22,27,32–35]. The formation of this chamber (Anfinsen’s cage) is the key structural argument for a suggestion that it is the place where the nonnative protein target bound with GroEL adopts the native structure in the absence of unfavorable contacts with other cellular components. This model of GroEL assisted protein folding has been worked out by several research groups during the recent 10 years and is widely accepted now [36–40]. Schematically the mechanism of protein folding in the GroEL inner cavity may be represented by the following main steps (Figure 2):

1. Nonnative protein binds with the preexisting asymmetrical complex GroEL:GroES in the inner cavity at the level of apical domains of the GroEL ring (*trans* ring) opposite to the ring bound to GroES.
2. Binding of the nonnative protein target induces an allosteric conformational change in the opposite ring leading to dissociation of GroES from GroEL.
3. GroES together with ATP binds once again with the GroEL ring containing nonnative protein (*cis* ring) and forms the so-called “Anfinsen’s cage” preventing unfavorable contacts of the protein substrate.
4. Cooperative hydrolysis of 7 ATP molecules by the *cis* ring enhances its affinity to GroES and leads to some conformational changes in apical domains resulting in release of the nonnative protein target into the “Anfinsen’s cage” for spontaneous folding in a state isolated from external medium.
5. Seven ATP molecules bind to the opposite “Anfinsen’s cage” ring (*trans* ring).
6. Cooperative hydrolysis of ATP by the *trans* ring leads to some allosteric conformational changes in the *cis* ring, which result in the dissociation of GroES and release of the native (folded) protein from the “Anfinsen’s cage”.

If one cycle is not enough for the final folding of the protein target, it reoccurs once again. Thus GroEL functions as a “molecular machine” controlled by its ligands, which induce allosteric conformational changes of the GroEL particle and define stages of the reaction cycle. This mechanism and its various modifications have so far undergone numerous experimental examinations and many experimental data have been satisfactorily explained within the framework of this model. At the same time, there are a number of experimental data showing that this mechanism of protein folding in the GroEL inner cavity is not universal yet. The present review is concentrated mainly on the data which are hardly intelligible supposing that GroEL-assisted protein folding occurs only by the above mechanism. An alternative mechanism of GroEL-assisted protein folding by the ligand-regulated binding-release of protein substrates and their folding in bulk solution is also discussed.

## 2. GroEL Assists Folding of Large Proteins Whose Size Exceeds the Chaperonin Inner Cavity

It is well established that GroEL is able to bind a wide range of nonnative or denatured proteins, as well as polypeptides of various sizes from 2 kDa up to more than 100 kDa both *in vitro* and *in vivo* [25,31,41–43]. The work of Houry *et al.* is an excellent example of identification of newly translated polypeptides tightly interacting with GroEL *in vivo* [25]. Using pulse radiolabelling, immunoprecipitation of GroEL with bound polypeptides, 2-D gel electrophoresis analysis and trypsinolysis combined with mass spectrometry, the authors demonstrated that ~ 20% of polypeptides bound with GroEL have molecular mass exceeding 60 kDa. In particular the list of these high molecular mass polypeptides includes phosphate acetyltransferase (77 kDa), tetrahydropteroyl-triglutamate methyltransferase (84 kDa), an RNA polymerase β-chain fragment (150 kDa), and DNA gyrase subunit A (97 kDa). The action of GroEL on the refolding of large proteins has not been thoroughly examined. The nonnative form of tailspike protein of phage P22 (70 kDa) interacts with GroEL both *in vitro* [44] and *in vivo* [45]. At elevated temperature (35 ºC) in the absence of ATP, GroEL effectively trapped protein refolding and reconstitution. However tailspike protein was released from GroEL by addition of ATP or without added ATP upon cooling to 25 ºC, and native tailspike trimers were formed. The presence of GroES in addition to GroEL had no effect on reconstitution yield [44]. The 86 kDa αβ heterodimer that is proposed to be an assembly intermediate of the α_2_β_2_ E1 enzyme of ketoacid dehydrogenase interacts with GroEL with 1:1 stoichiometry. The addition of Mg-ATP and GroES results in recovery of the active E1 tetramer [46]. The mechanism of GroEL-mediated folding of proteins, which are too large to be encapsulated under the GroES lid, is proposed for a large mitochondrial enzyme aconitase [47].The mitochondrial aconitase has molecular mass of 82 kDa that is too large to be encapsulated in the inner cavity of GroEL under the GroES lid, but the chaperonin assists the refolding of aconitase both *in vivo* and *in vitro*. The authors proposed that nonnative protein binds to the open (*trans*) ring of the GroEL-GroES complex and releases from the complex after binding and hydrolysis of ATP in the *cis* ring. The folding of the protein occurs in the free state (in bulk solution), and if the protein does not fold the cycle is repeated. The same mechanism was proposed for GroEL/GroES-assisted refolding of maltodextrin glucosidase (69 kDa monomeric *Eshcherichia coli* protein) [48].To check the possibility for the protein to be bound to the *trans* ring of GroEL, the opposite ring was connected with GroES through a flexible polypeptide linker that was short enough to prevent the protein penetration into the “Anfinsen’s cage” [49]. Nevertheless such an artificial GroEL-GroES system assists aconitase refolding with the same efficiency as the wild type system GroEL + GroES. Surprisingly this system revealed refolding assistance also for polypeptides of less molecular mass such as bacterial RuBisCo (~ 50 kDa) and mitochondrial malat dehydrogenase (~ 30 kDa). These proteins were proposed to be the so-called “stringent” substrates of GroEL, the productive folding of which requires encapsulation in the “Anfinsen’s cage” [50,51]. From these experimental data it is possible to conclude that the GroEL ring opposite to the GroES bound one can assist as well the folding of not only large proteins but also proteins which can be encapsulated in the chaperonin cavity.

## 3. GroEL Is Able to Assist Protein Folding in the Absence of GroES, ATP Hydrolysis and Double Ring Structure

GroEL functioning as a molecular chaperone is provided by interaction with a number of ligands: K and Mg ions, adenine nucleotides (ADP and ATP) and co-chaperonin GroES [10,12,18,29,31,32, 50,52]. However, it is impossible to make a general conclusion on the ligand involvement in the functioning of GroEL. The presence of GroEL either alone or in addition to ADP or ATP can be enough for effective GroEL-assisted protein folding [13,53–56], while the interaction with GroES usually enhances this reaction [54,57,58]. It seems likely that the ligands decrease the affinity of GroEL to nonnative protein targets with the efficiency increasing pursuant to Mg-ADP < Mg-ATP < Mg-ATP-GroES [58]. Studies of the protein refolding kinetics in the presence of GroEL and its ligands show that the chaperonin interacts with early kinetic intermediates having properties of the “molten globule” [19,59–61] and slows down their transition to the native state [58,62,63]. This interaction occurs very fast [62–64] and probably depends on the “hydrophobicity” and charge of the protein target [23,64–69] as well as on experimental conditions. If the lifetime of the GroEL complex with early protein refolding intermediates is comparably short (the so-called “transient interaction”), the refolding of the intermediates can be observed in the absence of ligands [62–64,70]. However, if the interaction of these intermediates with GroEL is strong (the lifetime of the complex is large), their refolding to the native state seems to be “arrested” during the observation time and appears only after an addition of GroEL ligands (see, for example, [50,71]), which likely decrease the GroEL affinity to protein refolding intermediates, and they can adopt native structure during the observation time. The double ring structure of GroEL is not required for assisting the protein folding as well. GroEL single ring conformation [72,73] and even isolated apical domains [74] are able to assist the protein folding. The mammalian mitochondrial chaperonin Hsp60 exists in a single ring conformation [75] but facilitates the refolding of ribulose-biphosphate carboxylase in an ATP-dependent manner [76,77]. The GroEL mutant with four amino acid substitutions in the equatorial interring surface also exists in a single ring conformation (SR1), but is functionally inactive probably due to its inability to release GroES [40,72]. However, the change of experimental conditions [57] or the change of mutations [78] lead to activation of the GroEL single ring conformation toward assisting the refolding of some proteins. Moreover, the 34 kDa proteolytic fragment of GroEL (residues 150 – 456) as well as the 50 kDa fragment of the *Thermus thermophilus* chaperonin are able to assist refolding of denatured rhodanese in the absence of GroES and ATP [79,80]. Fersht with co-workers constructed plasmids encoding the GroEL apical domain alone (residues 191 – 376) and some of its fragments and expressed them in *E. coli* cells [74]. They found that the GroEL apical domain alone and especially its C-terminal truncated fragments assist the refolding of rhodanese and cyclophilin, functioning as “minichaperones” in the absence of ATP. It may be suggested from the above experimental data that the inner cavity of GroEL is not directly related to its chaperoning activity. At the same time there are many evidences that the inner cavity participates in the GroEL-assisted protein folding (see reviews [10,12,81,82]). From recent publications it is possible to indicate the following:

Tang *et al.* [83] modulated the volume of the GroEL central cavity by a step-wise extension or reduction of the C-terminal sequence protruding from the equatorial domains into the cavity. They found that a change of the cavity volume resulted in both a change in the capacity of protein “encapsulation” and a change of the target protein refolding rate. Moreover, in a later publication they showed *in vivo* that a change of the GroEL cavity volume or charge affects cell viability and intracellular protein folding [84]. On the other hand, Farr *et al.* [85] reported that the effect of the GroEL C-terminal sequence extension on the protein folding rate may be due to the change in the ATP-ase activity. In addition the influence of the GroEL C-terminal sequence length on the chaperonin structure and its affinity to the substrate proteins can not be excluded either (our comment). Another set of the evidences goes from the mass-spectrometry and electron microscopy data indicating the place of target protein within the GroEL particle. Ester van Duijn *et al.* performed a number of mass-spectrometry studies of macromolecular complexes involved in the chaperonin-assisted protein folding. They found that the complex GroEL/protein substrate/co-chaperonin contains one GroEL particle, one monomeric protein substrate molecule and one co-chaperonin molecule [34]. However, they were unable to distinguish where the substrate molecule was bound either in the *cis* or *trans* ring of the complex. In another work the immersion of some substrate proteins into the GroEL particle was studied using native mass spectrometry combined with ion mobility mass spectrometry (IM-MS) [35]. It was shown that the complexes of GroEL with a number of denatured protein substrates have dimensions (“collision cross sections”) similar to those of free GroEL which shows that these genuine substrates are buried within the chaperonin cavity. In contrast, the binding of native BSA to GroEL resulted in a significantly larger dimension of the complex in comparison with free GroEL. The authors concluded that the binding of the native BSA ligand to GroEL is nonspecific and occurs on the top of the apical domains out of the cavity. Using the same approach the authors showed that the binding of GroES to the pre-existing GroEL/protein substrate complex increases the complex dimension (“collision cross section”) only by the amount of GroES, confirming deep immersion of the protein substrate into the cavity [35].

A more obvious location of the substrate protein molecule within the GroEL/co-chaperonin complex was confirmed by cryo-electron microscopy. However, the data obtained for different chaperonins and protein substrates are somewhat conflicting. In early publications it was reported that the non-native protein substrate is placed in the *trans* ring of the GroEL-GroES complex for both *Escherichia coli* [22] and *Thermus thermophilus* [27] chaperonin systems. Recent publications show that the protein substrate molecule is placed in the *cis* ring of the GroEL/co-chaperonin complex for both *Escherichia coli* GroEL with the bacteriophage T4 GroES – analog (gp31) system [33] and *Thermus thermophilus* GroEL-GroES system [86]. This contradiction may be a result of different techniques used for preparation of the samples, or the co-chaperonin can distinguish what GroEL ring must be the *cis* one in dependence on the substrate protein. In any case, the electron microscopy data give us visual information that the protein substrate can occupy both GroEL rings simultaneously and can be encapsulated in the cavity [33,86].

The importance of the ring-like organization of the GroEL particle and hence its inner cavity is somewhat supported by the data that the protein target can undergo multiple partial unfolding to overcome misfolded conformations and enhance the yield of a correct folded conformation in accordance with “the iterative annealing mechanism” [87]. However this mechanism is still argued due to experimental difficulties to distinguish between protein substrate unfolding as a result of its binding to GroEL and as a result of the shift of the equilibrium toward a more-unfolded protein state that may preferentially bind to GroEL (see the discussion in the review by Horwich *et al.* [12]).

## 4. GroEL Is Able to Bind Simultaneously More Than One Polypeptide Substrate or Two GroES Molecules

The stoichiometry of the GroEL-polypeptide complex is poorly studied and the existing data are again conflicting. The crystal structure of GroEL with a bound small polypeptide (~ 2 kDa) in the absence of nucleotides shows that small polypeptides bind to each of 14 subunits in the region of a pair of parallel helices at the top of the apical domain [41]. The same polypeptide binding sites have been found in the isolated apical domain of GroEL [74,78]. Interestingly, the polypeptides are bound to GroEL at the same places as the GroES molecule [32,41]. As for large polypeptides such as proteins, the data are contradictory. Titration calorimetry experiments on the GroEL binding with two denatured proteins (pepsin at pH 7 and reduced α-lactalbumin) show 1:1 stoichiometry for the both proteins [88]. The equimolar stoichiometry of the nonnative protein target bound to the GroEL particle was reported also for a mutant form of subtilisin [89]. However, for maltose-binding protein [55] and for mutants of staphylococcal nuclease [90] the stoichiometry of the GroEL/substrate complex was demonstrated to be 1:2. GroEL was reported to bind up to 4–5 molecules of nonnative barnase [91] or mutant forms of dihydrofolate reductase [92]. Chuang and co-workers reported that GroEL saturated with a nonnative αβ heterodimer of α-ketoacid dehydrogenase up to 1:1 stoichiometry is able to bind additionally a stoichiometric amount of denatured lysozyme [46]. Young and co-workers have shown that integral membrane proteins can be soluble without any detergent by binding to the GroEL tetradecameric particle. In particular, after dialysis of detergent-solubilized bacteriorhodopsin in the presence of GroEL, it was found that GroEL binds two molecules of the protein at saturation [93]. In the other study they analyzed solubilization of a membrane protein λ-holin by GroEL and found that the protein saturates the chaperonin at six molecules per tetradecameric GroEL particle, whereas the protein variant missing two N-terminal residues forms a hypersolubilization complex with up to 350 holin molecules per GroEL particle. In the presence of ATP or its nonhydrolyzed analog AMP-PNP no solubilization was observed, while in the presence of ADP slow precipitation of holin molecules was detected [94]. Stoichiometry measurements of GroEL-protein target complexes may be complicated by polypeptide impurities tightly bound with GroEL [95]. We purified GroEL from such impurities according to the published protocol [96] and measured the stoichiometry of GroEL complexes with denatured pepsin, reduced α-lactalbumin, lysozyme and bovine serum albumin using fluorescence anisotropy titration and size-exclusion chromatography. Two protein molecules per GroEL tetradecamer were found to be bound for all of these proteins (our unpublished data). The data from mass spectrometry [34] and cryo-electron microscopy [33] also show 1:2 stoichiometry of the GroEL/protein substrate binary complex. The symmetrical GroEL double ring structure is able to bind not only more than one polypeptide target but under certain conditions two molecules of its co-chaperonin GroES [97–102]. Thus, the GroEL complex with GroES can exist in two forms: asymmetrical and symmetrical [98,99].

## 5. GroEL-GroES Complex Does Not Maintain Interactions with Denatured Proteins

Most experiments on the release of target proteins from the GroEL-GroES complex are performed using protein refolding systems. However, in such cases it is difficult to distinguish the events of formation of protein native structure and protein dissociation from the complex because native protein usually does not interact with GroEL. However, if the formation of protein native structure is inhibited by experimental conditions (the presence of disulfide bond reducing agents, nonnative pH or temperature) or by mutations or truncation of the protein chains it is possible to study more easily the binding and release of protein targets. Using size-exclusion chromatography Yoshida and co-workers showed that GroEL in the absence of its ligands tightly binds pepsin denatured at neutral pH. Mg-ATP decreases GroEL affinity to the protein target while the binding of GroES in the presence of Mg-ATP leads to practically full dissociation of denatured pepsin from the GroEL-GroES complex [88]. A similar result was obtained by us later [67]. Moreover, in some cases when denatured proteins are strongly aggregated, the GroEL-GroES complex is not able to prevent their aggregation as it was shown by us for reduced lysozyme [103] and for truncated variants of elongation factor EF-2 [104]. These data challenge the proposition that nonnative proteins of appropriate molecular mass must be enclosed in the *cis* chamber of the GroEL-GroES complex. Indeed, the timing of a GroEL/GroES assisted protein folding reaction shows that at 23 ºC the polypeptide bound with GroEL is released into the closed chamber within a second after a *cis* complex formation [105]. The substrate protein then lives about 10 s in the cavity under GroES lid till GroES can be discharged by the binding of ATP in the *trans* ring (Figure 2) [101,106,107]. Thus, the equilibrium must be strongly shifted towards the GroEL/substrate protein/GroES ternary complex. In accord with this, if protein target cannot adopt the rigid (native) structure it must be mainly involved in the ternary complex and cannot be separated from the complex by size-exclusion chromatography [67,88] or aggregated [103,104]. On the contrary, these data support the proposal that interaction of GroES with the binary GroEL-nonnative protein complex decreases the affinity of the chaperonin to the protein target may be due to the competition between co-chaperonin and nonnative proteins for the same binding sites.

## 6. The Models of GroEL-Assisted Protein Folding out of the Chaperonin Inner Cavity

**Figure 3.**
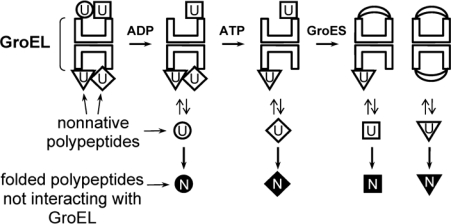
Scheme illustrating the mechanism of GroEL functioning as a molecular chaperone under the assumption of the target protein folding in the free state (in bulk solution) [108]. Different symbols indicate different polypeptides which are bound by GroEL with different association constants. Nonnative (unfolded) and native conformations of polypeptides are indicated as U and N, respectively.

The experimental data reviewed herein are hardly understandable assuming that the protein target can be folded only in the inner cavity of GroEL under the GroES lid. Therefore some investigators suggest that GroEL assists large proteins folding by a “*trans*” mechanism [47–49]. This mechanism proposes that protein folding occurs in bulk solution while the release of the protein target from GroEL is due to allosteric conformational changes in the “*trans*” ring bound with protein substrate which are induced by GroES binding in the “*cis*” ring. Recently we proposed some model by which GroEL may assist the folding of various proteins by time- or ligand-controlled multiple rounds of binding-release of the protein targets and their final folding in bulk solution [108] (Figure 3).

The model is based on the following principles: The GroEL particle is able to bind various polypeptides lacking rigid tertiary structure on both of its barrel ends including the tops of the cavities. The number of bound polypeptides depends on their size while the affinity to GroEL is determined by their hydrophobicity and charge. Thus, the concentration of nonnative polypeptides in bulk solution (in the free state) drops abruptly which reduces the probability of their nonspecific interactions. Weakly bound polypeptides can sometimes dissociate from the GroEL surface and adopt native (rigid) conformation in bulk solution or interact with other cellular factors even in the absence of GroEL ligands.

The ligands (Mg-ADP, Mg-ATP and co-chaperonin GroES) weaken to a different extent the interaction of nonnative polypeptides with GroEL. As a result the lifetime of polypeptides in the free state and hence the probability for them to adopt native structure or to be bound to other cellular factors preventing their nonspecific association (e.g., to other chaperones) is enhanced.

The model does not require the protein target encapsulation in the “Anfinsen’s cage” where its folding may be problematic and does not impose strict limitations on the target size and stoichiometry of the complexes with GroEL. At the same time such a mechanism provides the GroEL function to prevent nonspecific intermolecular association of polypeptides lacking a rigid tertiary structure. Thus, GroEL functioning as a molecular chaperone is restricted by binding strongly “hydrophobic” states of polypeptides resulting in lowering their concentration in bulk solution and assisting their folding through ligand- or time-dependent multiple binding-release rounds. GroEL ligands prevent formation of long-living chaperonin-substrate complexes which are not advantageous for fast recovery of cells after the stress.

## 7. Conclusions

It is now well known that protein (re)folding is often complicated by nonspecific intermolecular interactions of protein molecules in intermediate states with high exposure of hydrophobic groups. These interactions may lead to a slowing down the protein native structure formation or, under strongly nonpermissive conditions, to the formation of large aggregates which practically inhibit protein folding. Nonspecific intermolecular interactions upon protein folding *in vitro* can be essentially reduced by choosing appropriate refolding conditions. Among them the following seems to be most effective. The first is temperature drop leading to weakening of hydrophobic attraction. The second is a decrease of the protein concentration that reduces the probability of intermolecular association of protein molecules. The third is enhancement of the electrostatic repulsion of aggregation-prone intermediates that can be achieved either by decreasing ionic strength of the renaturing mixture or by changing pH. In some cases the addition of denaturants (urea or GuHCl) or low molecular weight compounds (such as arginine, glycerol, sucrose) to the renaturing mixture is also effective. Very often these simple operations allow overcoming the problems accompanying protein folding *in vitro*. However, in the cell the protein aggregation is controlled by chaperones. The majority of chaperones is able to recognize polypeptides lacking rigid tertiary structure and to prevent their nonspecific association (see, for example, [10,11,13]). Many chaperones work using the ligands which provide regulation of binding/release rounds of the substrate polypeptides. Among them there are monomeric or dimeric chaperones with hydrophobic pockets for substrate polypeptides binding as well as more complex oligomeric chaperones which bind polypeptide substrates by multivalent interactions with exposed hydrophobic clusters of their subunits [11]. The quaternary structure of the latter is usually formed as one or two ring-shaped oligomers consisting of several homo- or hetero-subunits forming a distinct inner cavity. Despite of similar organization of the quaternary structure of these chaperones and the presence of inner cavity, the interaction with GroES-like co-chaperones has been found only for GroEL-like representatives of this group of chaperones. The inner cavity of chaperones lacking co-chaperones seems to be less essential for protein folding than in the case of GroEL. Indeed a partial occlusion of both cavities in the eukaryotic chaperonin CCT with antibody has no effect on the rate and yield of substrate protein folding [110]. A variety of chaperones may be divided according to the mechanisms of their functioning in the following groups. The first group includes small heat shock proteins and α-crystalline which prevent protein aggregation in an ATP-independent manner [111,112]. These oligomeric proteins, consisting of 12 – 43 kDa subunits with different multimeric organization, bind denatured proteins but their release from small heat shock proteins is very slow. It has been proposed that their main function is to prevent aggregation of denatured proteins and to form some reservoir for other chaperone systems to renature the bound proteins [111]. Small heat shock proteins do not show substrate specificity and possibly bind polypeptide substrates on the external surface of the multimer [11]. The second group represents monomeric and dimeric chaperones whose functioning is dependent on the ADP/ATP ligands and protein cofactors. Molecular chaperones of the Hsp70 family not only bind substrate proteins and prevent their aggregation but provide their subsequent folding (see reviews [10,11]). They are composed of two functional parts one of which binds ADP or ATP and possesses ATP-ase activity, whereas the other one binds substrate polypeptides. Chaperones of this family are usually functioning in cooperation with two chaperones of the Hsp40 family [10]. ATP binding induces some conformational changes in Hsp70 resulting in decreasing the chaperone affinity for nonnative substrate proteins [113]. Polypeptide substrates released from the Hsp70 chaperones undergo kinetic partitioning between folding to native conformation, aggregation, rebinding to the chaperone and binding to other components of a multidirectional protein folding network. Similar activities have been shown for dimeric chaperones of the Hsp90 family [11]. They also use adenine nucleotides and interaction with other protein cofactors and chaperones to provide effective protein folding. The third group of chaperones represents oligomeric ring-like organized complexes with distinct inner cavities. To execute their functions, these chaperones require adenine nucleotides and some of them also GroES-like co-chaperonins. Members of the Hsp100 family are six-subunit ring-like complexes which contain ATP and substrate polypeptide binding sites and are able to substitute the Hsp70 chaperone systems in effective protein refolding [11,114]. The other members of this group are the so-called chaperonins which are organized as multimeric double ring complexes with two extensive inner cavities. Among them it is possible to discriminate GroEL-like chaperonins having GroES-like co-chaperonins and those which have no co-chaperonins [11–13,15]. Nevertheless both types of chaperonins assist protein folding both *in vivo* and *in vitro* by multiple ATP-dependent repeated binding-release of substrate polypeptides [12]. Up to date only for the GroEL/co-chaperonin complex the substrate protein was found inside the cavity under the GroES lid. The above analysis of published data raises the question about the importance of inner cavities of oligomeric chaperones as the universal place where protein substrates are prevented from aggregation and can adopt native structure. Further studies of the properties of monomeric and oligomeric chaperones from different organisms and cellular compartments will allow us to clarify general principles of their participation in the processes of protein creation and transport in the cell.
